# The consumption of low-calorie sweetener containing foods during pregnancy: results from the ROLO study

**DOI:** 10.1038/s41430-021-00935-0

**Published:** 2021-05-26

**Authors:** Marie C. Conway, Shona Cawley, Aisling A. Geraghty, Niamh M. Walsh, Eileen C. O’Brien, Fionnuala M. McAuliffe

**Affiliations:** UCD Perinatal Research Centre, School of Medicine, University College Dublin, National Maternity Hospital, Dublin, Ireland

**Keywords:** Public health, Nutrition, Health care

## Abstract

**Background/objectives:**

Women with gestational diabetes (GDM) are advised to adapt a low glycaemic index (GI) diet, which may impact consumption of low-calorie sweeteners (LCS). LCS are increasingly popular as they add sweetness without contributing calories. This study aims to investigate the reported intakes of LCS-containing foods in women during pregnancy.

**Subjects/methods:**

Pregnant women recruited for the ROLO study were included in this analysis (*n* = 571). Women were randomised to receive either an intervention of low-GI dietary advice or usual antenatal care. Women completed a 3-day food diary in each trimester. Nine LCS-containing food groups were identified, and the quantity (g/day) consumed was calculated.

**Results:**

One-third of all pregnant women consumed LCS across each trimester of pregnancy. Of those in the intervention group who were LCS consumers in trimester 1, 71.6% were consumers in trimester 2, and 54.1% remained consumers in trimester 3. In the control group, less women remained consumers in trimester 2 and 3 at 58.1% and 41.9%, respectively. In trimester 2, following the dietary intervention, the proportion of LCS consumers in the intervention group was significantly higher than the proportion of consumers who were in the control group (*p* < 0.001). The most commonly consumed food groups were low-calorie fruit drinks, diet-cola drinks, and low-calorie yoghurts.

**Conclusions:**

One-third of pregnant women consumed LCS. The proportion of LCS consumers increased in the intervention group compared to the control group. Further research is needed to determine exposure levels to individual LCS, and the effect of prenatal exposure to LCS on maternal and child health outcomes.

## Introduction

Low-calorie sweeteners (LCS) are substances added to foods and beverages to add a sweet taste, without adding calories [1]. Consumption of LCS has become increasingly popular due to their low-calorie content [2]. Globally, obesity and its associated comorbidities are amongst the most important public health concerns. In developed countries, overweight or obesity affects one-third of children and two-thirds of adults, with associated annual medical costs of €33 billion in the European Union [3]. Added sugar intake is strongly associated with obesity and related comorbidities, prompting population-wide recommendations to reduce sugar consumption [4]. The consensus from the scientific evidence and the World Health Organisation (WHO) is to reduce intake of free sugars to below 10% of total energy intake, and the WHO has recommended to further reduce free sugars to less than 5% of total energy intake [5]. Despite the increased prevalence of LCS consumption [2, 6], little is known about their consumption during pregnancy. There is a body of literature that suggests chronic LCS consumption may increase the risk of obesity and metabolic diseases [7]. Proposed mechanisms for this association include alteration of glucose metabolism [8], disruption of gut microbiota [9], or dysregulation of satiety and caloric compensation [10]. This evidence has, however, been generated in adult studies and there is a dearth of data on consumption and its effect on metabolic health in pregnancy. LCS have been shown to have an influence on the gut microbiota [11]. The maternal microbiome is important in determining infant microbiome [12]. Therefore, changes in maternal microbiome due to LCS may in turn influence fetal programming, specifically the microbiota of the infant. Some research has been conducted investigating the effects of LCS consumption during pregnancy on child outcomes, however, this has focused on single foods, not multiple food groups [13]. The effect of LCS intake in children on weight gain, fat mass accumulation, and body mass index (BMI) has yielded inconsistent results [3]. Prenatal exposure to LCS and child health outcomes warrants further investigation; however, before these associations can be investigated, knowledge of LCS dietary intake across various population groups is necessary.

It is worth noting that all LCS undergo toxicological evaluation prior to approval at national and international levels, which considers reproductive toxicology and exposure during pregnancy and early life [14]. As part of this evaluation, an acceptable daily intake (ADI) is developed. The ADI is an estimation of the amount of LCS that can be consumed daily by an individual over a lifetime without an appreciable risk to health [15]. The US Academy of Nutrition and Dietetics states that consumption of LCS within the stated ADI is safe in pregnant women and in young children [16]. Conversely, the Institute of Medicine argues that there is a lack of evidence on the long-term health effects of the use of LCS when used from early childhood [17]. In Europe, recent studies have shown that LCS consumption is much lower than the ADI in adults and children, even in those who are categorised as ‘high consumers’ [18]; however, pregnant women were not included in these studies. Before research can be conducted on long- and short-term effects of early exposure to LCS, it is important firstly, to establish the levels at which pregnant women consume LCS.

In a recent study, it was found that approximately a quarter of pregnant women in the United States were consuming LCS as assessed using data from 1999 to 2014 [2]. Consumption of LCS has been increasing across this time period, rising from 16.2% in 1999–2004 to 24% in 2007–2014. The highest prevalence of LCS consumption amongst pregnant women was observed in 2005–2006 with 38.4% consuming LCS. This increased prevalence is reflective of an increase in the proportion of pregnant women consuming LCS beverages over the same time period from 9.9% in 1999–2004, to 18.3% in 2007–2014 [2]. Consumption was found to be higher among married women than those who were not married, and the prevalence of intake increased with maternal age [2].

Globally, women with gestational diabetes mellitus (GDM) are recommended to follow a low glycaemic index (GI) diet, which involves exchanging high-GI carbohydrates for low-GI alternatives [19]. Therefore, a low-GI diet may impact on the levels of LCS consumed by pregnant women, and likely reflects the change from sugar to sweeteners among women diagnosed with GDM. It is necessary to understand consumption levels of LCS in pregnant women and how these compare to consumption in the wider population. This will in turn allow for further research to be conducted to elucidate the long-term health effects of consumption of LCS during pregnancy which will have impact on public health guidance by informing dietary recommendations for expectant mothers worldwide. The current study aims to investigate the reported intakes of LCS-containing foods during each trimester of pregnancy, and to determine if there are differences in reported consumption of LCS-containing foods between women in the control and intervention group of a low-GI dietary intervention.

## Materials and methods

### Study design and population

This is a longitudinal study of 571 women originally recruited as part of the Randomised cOntrol trial of LOw glycaemic index diet in pregnancy to prevent recurrence of macrosomia (ROLO study) at The National Maternity Hospital, Dublin, Ireland (ISRCTN54392969) [19]. Women were included in the current analysis if they had completed a food diary at each trimester of pregnancy. Recruitment for this study was carried out between 2007 and 2011, as previously described [19]. In summary, secundigravida women who had previously delivered a macrosomic infant (>4000 grams) were recruited at their first antenatal appointment and subsequently randomised to receive either low-GI dietary advice or to the control group of usual care (no dietary advice) [19]. For those in the control group, women received routine antenatal care, which did not involve any specific advice about gestational weight gain or formal dietary advice [19]. Women randomised to the intervention group attended a 2-hour group education session with a research dietitian in groups of two to six women. This took place 2 weeks post randomisation, at approximately 15 weeks’ gestation. Women were taught the principles of healthy eating, including guidelines for pregnancy and the food pyramid. Information on GI, including what this is, and the rationale for use in pregnancy was also included in the session, and women were given advice on how to follow a low-GI diet in pregnancy. As outlined previously, it was recommended to women that they choose low-GI foods where possible, and they were also provided with written resources after the session [19]. The research dietitian met with intervention subjects at 28- and 34-weeks’ gestation for brief reinforcement of the diet, and to answer any questions they may have had. Institutional ethical approval was granted and all mothers provided written consent at the National Maternity Hospital, Dublin.

### Anthropometry and lifestyle information

Maternal height and weight were measured in early pregnancy (13 weeks’ gestation). BMI (kg/m^2^) was subsequently calculated. Gestational weight gain was calculated by subtracting the measured weight at the first antenatal visit from the final weight in pregnancy. Data were collected on maternal age, ethnicity, parity, maternal educational attainment, and smoking status.

### Dietary intakes and LCS consumption

Dietary intakes in pregnancy were collected using 3-day food diaries [20, 21]. All food and beverages consumed over 3 consecutive days were recorded by participants during each trimester, with women encouraged to include one weekend day. Women were asked to quantify foods consumed using the weight provided by the manufacturer on food packaging or using standard household measures, for example, tablespoons. In cases where the amount consumed was not clearly recorded, average portion sizes according to the Food Standards Agency [22] were used, or the researcher estimated this based on the participant’s eating patterns [21]. Dietary data were entered into dietary analysis software NETWISP version 3.0 (Tinuviel Software, Llanfechell, Anglesey, UK). The NETWISP food composition database was derived from the 6th edition of McCance and Widdowson’s food composition tables [23]. Food codes included in NETWISP were grouped into containing LCS sweetener or not. Foods were included if they contained the terms “diet”, “no added sugar”, “diabetic” among others. Nine LCS-containing food groups were identified: instant hot chocolate powder, ice cream, diabetic chocolate, diet cola, diabetic spread, low-calorie soup, low-calorie salad cream, low-calorie yoghurts, low-calorie fruit drinks. Consumption of these foods was then analysed. The quantity of each LCS-containing food group consumed by each participant was recorded as grams per day (g/day). Consumption of table-top sweeteners was not recorded. If a woman consumed any one of the LCS-containing foods she was defined as a “consumer of LCS-containing foods”. If a woman did not consume any LCS-containing foods she was defined as a “non-consumer of LCS-containing foods”.

### Statistics

Statistical analysis was completed using Statistical Package for Social Sciences (SPSS) v24 (SPSS, IBM, Chicago, IL, USA) with statistical support provided by the Centre for Support and Training in Analysis and Research (CSTAR), University College Dublin (UCD). All variables were evaluated for normal distribution by visually analysing histograms. Descriptive statistics were used to determine the characteristics of the participants who provided food diaries (*n* = 571). Daily intakes of LCS-containing food groups were determined for pregnant women in each trimester. Differences between groups were investigated using independent sample *t*-tests for normally distributed data, or Mann Whitney-U for non-normal data. Chi-squared tests for independence were used to examine differences in categorical variables. A *p* value of <0.05 was considered statistically significant.

### Results

Of the 800 women originally randomised to take part in the ROLO trial, a total of 571 women were included in the current analysis. Maternal characteristics are detailed in Table 1. The median age of the women was 32.9 years old, with a median BMI of 25.5 kg/m^2^, which is in the overweight category. Of the women included in the current analysis, almost half (*n* = 258; 45.2%) were originally randomised to the intervention group and the remainder were randomised to the control group (*n* = 313; 54.8%). The majority were Caucasian (97.9%). In total, 56.1% had completed third level education. The number of pregnant women who were consumers of LCS in each trimester is also shown in Table 1, indicating a slightly higher proportion of LCS consumers in trimester 3 (34.0%), than in trimester 1 (29%.2) or trimester 2 (32.3%).

**Table 1:** Maternal demographic characteristics of pregnant women with food diary data.

	*n*	Median (IQR) or *n* (%)
Maternal age at delivery (years)	571	32.9 (30.2, 35.4)
Maternal BMI (kg/m^2^)^a^	568	25.5 (23.3, 28.4)
Gestational weight gain (kg)	477	13.0 (10.4, 15.7)
Energy Intake (kcal/day)		
Trimester 1	548	1816.3 (1572.1, 2109.0)
Trimester 2	554	1845.9 (1575.0, 2137.3)
Trimester 3	549	1847.7 (1594.7, 2165.9)
Intervention vs. Control^b^	571	
Intervention		258 (45.2)
Control		313 (54.8)
Ethnicity^b^	571	
Caucasian		559 (97.9)
Other		12 (2.1)
Education Level^b^	542	
Some secondary		24 (4.4)
Complete secondary		87 (16.1)
Some 3rd level		127 (23.4)
Complete 3rd level		304 (56.1)
Current smoker^b^	541	
Yes (regularly)		23 (4.3)
Yes (occasionally)		14 (2.6)
No		504 (93.2)
LCS consumers^b^		
Trimester 1	547	
Consumers		160 (29.3)
Non-consumers		387 (70.7)
Trimester 2	554	
Consumers		179 (32.3)
Non-consumers		375 (67.7)
Trimester 3	549	
Consumers		187 (34.1)
Non-consumers		362 (65.9)

*IQR* Interquartile range (expressed as 25th and 75th centile), *BMI* body mass index, *LCS* low-calorie sweeteners.

^a^BMI measured in trimester 1.

^b^Indicates data expressed as *n* (%).

Given that not all pregnant women were consumers of LCS, the general characteristics of the cohort were examined according to whether they were “consumers” or “non-consumers” of LCS as shown in Table 2. Potential differences in energy intake in each trimester between consumers and non-consumers were analysed. There was no significant difference in energy intake between consumers and non-consumers, with this being the case in each of the trimesters. Maternal BMI was significantly lower in non-consumers (*p* = 0.043). In trimester 2, the proportion of LCS consumers in the intervention group was significantly higher than the proportion of consumers who were in the control group (*p* < 0.001). Of those in the control group who were consumers of LCS in trimester 1 (*n* = 86), 50 (58.1%) remained consumers of LCS in trimester 2, and 36 (41.9%) continued to be consumers in trimester 3 (Supplementary Table 1). In trimester 2, a further 27 women in the control group who had not been consumers in trimester 1 became consumers, and 34 women who had not been consumers in trimester 1 and 2 became consumers in trimester 3. In the intervention group, of those who were consumers of LCS in trimester 1 (*n* = 74), 53 (71.6%) remained consumers of LCS in trimester 2, and 40 (54.1%) remained consumers of LCS in trimester 3. A total of 49 women in the intervention group who were non-consumers in trimester 1 began consuming LCS in trimester 2, and a further 17 became LCS consumers in trimester 3 (Supplementary Table 1).

**Table 2:** Characteristics of consumers vs non-consumers of low-calorie sweeteners (LCS).

	Consumers (*n* = 287)		Non-consumers (*n* = 284)		*p* value
	*n*	Median (IQR)	*n*	Median (IQR)	
Maternal age at delivery (years)	287	33.0 (30.6, 35.4)	284	32.7 (29.9, 35.4)	0.490
Maternal BMI (kg/m^2^)^a^	284	25.9 (23.5, 28.6)	284	25.3 (23.0, 28.2)	0.043
Gestational weight gain (kg)	243	13.2 (10.4, 15.6)	234	12.9 (10.3, 15.8)	0.472
Energy intake (kcal/day)					
Trimester 1	160	1820.7 (1615.5, 2144.5)	387	1812.7 (1544.8, 2107.6)	0.359
Trimester 2	179	1847.5 (1572.5, 2076.8)	375	1844.2 (1575.9, 2164.6)	0.676
Trimester 3	187	1838.5 (1609.4, 2129.4)	362	1866.3 (1585.0, 2202.7)	0.936
Intervention vs. control^b^					
Intervention		140 (48.8)		118 (41.5)	0.099
Control		147 (51.2)		166 (58.5)	
Ethnicity^b^					
Caucasian		284 (99.0)		275 (96.8)	0.140
Other		3 (1.0)		9 (3.2)	
Education level^b^					
Some secondary		8 (2.9)		16 (6.1)	0.169
Complete secondary		49 (17.6)		38 (14.4)	
Some 3rd level		70 (25.2)		57 (21.6)	
Complete 3rd level		151 (54.3)		153 (58.0)	
Current smoker^b^					
Yes (regularly)		10 (3.6)		13 (4.9)	0.734
Yes (occasionally)		7 (2.5)		7 (2.7)	
No		261 (93.9)		243 (92.4)	
Trimester 1^b^		160 (29.2)		387 (70.8)	
Intervention		74 (30.1)		172 (69.9)	0.771
Control		86 (28.6)		215 (71.4)	
Trimester 2^b^		179 (32.3)		375 (67.7)	
Intervention		102 (41.1)		146 (58.9)	<0.001
Control		77 (25.2)		229 (74.8)	
Trimester 3^b^		187 (34.0)		362 (66.0)	
Intervention		90 (36.7)		155 (63.3)	0.273
Control		97 (31.9)		207 (68.1)	

*p* value for significant difference between groups as determined using independent *t* test, Mann Whitney *U* test or Chi square where appropriate; *p* < 0.05 considered significant.

*IQR* Interquartile range (expressed as 25th and 75th centile), *BMI* body mass index, *LCS* low-calorie sweeteners.

^a^BMI measured in trimester 1.

^b^Indicates data expressed as *n* (%).

**Fig. 1:**
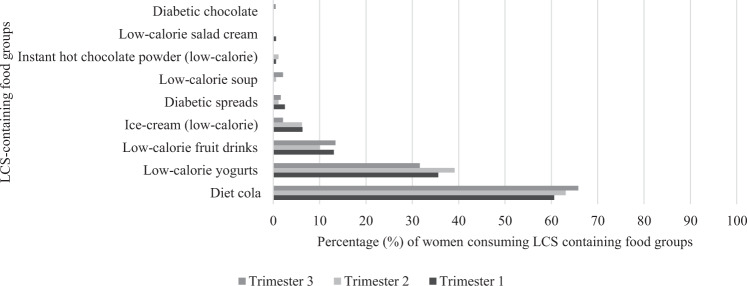
Graph showing the percentage (%) of women consuming food groups containing low-calorie sweeteners (LCS) in each trimester of pregnancy. Percentage (%) consumers for Trimester 1, Trimester 2, and Trimester 3 indicated according to shade of bar in graph.

The percentage of women consuming each of the LCS-containing food groups in each trimester of pregnancy is shown in Fig. 1 and indicates diet cola, low-calorie yogurts and low-calorie fruit drinks were most commonly consumed. Table 3 shows the nine LCS-containing food groups identified in the women’s food diaries and the amount of each consumed according to intervention or control group. Low-calorie fruit drinks, diet cola, and low-calorie yoghurts were the LCS-containing food groups consumed most frequently according to the number of people consuming these in each of the trimesters for both the intervention and control group. In trimester 1, there was a significant difference in the daily intake of low-calorie yogurts between the intervention and control group, with the intervention group consuming a higher amount. There was no significant difference in consumption of any of the other food groups between the intervention and control groups in any trimester.

**Table 3:** Dietary intake (g/day) of low-calorie sweetener (LCS) containing foods in each trimester of pregnancy according to RCT group for those who were consumers of LCS.

Food group	*n*	%	Mean	SD	Median	IQR	*n*	%	Mean	SD	Median	IQR	*p* value
	**Trimester 1 (*n*** = **160)**												
	**Intervention (*n*** = **74)**						**Control (*n*** = **86)**						
Diet cola	44	59.5	161.51	118.9	110	87.50, 189.33	53	61.6	204.56	184.88	126.67	110.00, 225.00	0.232
Low-calorie yogurts	30	40.5	73.17	32.7	83.33	41.67, 83.33	27	31.4	57.47	28.07	41.67	41.67, 83.33	0.036
Low-calorie fruit drinks	11	14.9	338.67	326.49	194.67	66.67, 568.00	10	11.6	404.33	359.76	300.00	111.67, 595.17	0.666
Ice-cream (low-calorie)	5	6.8	35.00	15.41	25.00	25.00, 50.00	5	5.8	48.00	21.68	50.00	30.00, 65.00	0.306
Diabetic spreads	1	1.4	5.00		5.00		3	3.5	8.33	2.89	10.00		0.317
Low-calorie soup	0	0.0					0	0.0					
Instant hot chocolate powder (low-calorie)	1	1.4	6.00		6.00		0	0.0					
Low-calorie salad cream	0	0.0					1	1.2	49.33		49.33		
Diabetic chocolate	0	0.0					0	0.00					
	**Trimester 2 (*n*** = **179)**												
	**Intervention (*n*** = **102)**						**Control (*n*** = **77)**						
Diet cola	58	56.9	192.38	139.70	166.67	83.33, 223.33	55	71.4	202.41	157.29	166.67	110.00, 220.00	0.437
Low-calorie yogurts	48	47.1	62.02	30.77	41.67	41.67, 83.33	22	28.6	58.48	28.77	41.67	41.67, 83.33	0.633
Low-calorie fruit drinks	10	9.8	651.67	628.06	429.33	189.33, 1008.67	8	10.4	364.17	415.55	194.67	66.67, 725.00	0.130
Ice-cream (low-calorie)	5	4.9	20.33	7.94	25.00	13.33, 25.00	6	7.8	37.11	27.65	25.00	23.75, 49.42	0.162
Diabetic spreads	1	1.0	4.00		4.00		1	1.3	15.00		15.00		0.317
Low-calorie soup	1	1.0	5.33		5.33		0	0.0					
Instant hot chocolate powder (low-calorie)	2	2.0	35.33	44.31	35.33		0	0.0					
Low-calorie salad cream	0	0.0					0	0.0					
Diabetic chocolate	0	0.0					0	0.0					
	**Trimester 3 (*n*** = **187)**												
	**Intervention (*n*** = **90)**						**Control (*n*** = **97)**						
Diet cola	56	62.2	181.98	148.69	110.5	86.33, 255.00	67	69.1	177.41	138.35	133.33	110.00, 220.00	0.736
Low-calorie yogurts	31	34.4	71.99	42.49	50.00	41.67, 83.33	28	28.9	66.07	39.76	41.67	41.67, 83.33	0.418
Low-calorie fruit drinks	15	16.7	366.53	380.18	189.33	66.67, 568.00	10	10.3	426.80	413.82	364.00	66.67, 637.50	0.910
Ice-cream (low-calorie)	4	4.4	31.3	12.5	25.00	25.00, 43.75	0	0.0					
Diabetic spreads	3	3.3	12.00	11.61	8.33		0	0.0					
Low-calorie soup	1	1.1	6.67		6.67	6.67, 6.67	3	3.1	5.00	2.89	6.67		0.564
Instant hot chocolate powder (low-calorie)	0	0.0					0	0.0					
Low-calorie salad cream	0	0.0					0	0.0					
Diabetic chocolate	1	1.1	5.00		5.00	5.00, 5.00	0	0.0					

Mean, SD, median and IQR values displayed are for grams/day amount of each food group consumed; IQR interquartile range expressed as 25th and 75th percentile; Trimester 1 intake reflects consumption before intervention began, and therefore before dietary information was given to women allocated to the intervention group; *p* value for significant difference between intervention and control groups in each trimester as determined using independent t test or Mann Whitney *U* test; *p* < 0.05 considered significant.

## Discussion

The current study presents data on the intakes of LCS-containing foods in pregnant women, identifying approximately one-third of these women as being consumers of LCS. In terms of demographics, rates of third level education (classified as attaining at least a level 8 college degree) were reported as 56.1% which is unsurprising as past research has shown that women with a higher level of education are more likely to take part in research [24]. In terms of consumers across the trimesters, there were 160 (29.2%) reported consumers of LCS in trimester 1, 179 (32.3%) in trimester 2, and 187 (34.0%) in trimester 3. This indicates a slight increase in the number of consumers as pregnancy progresses. Also, non-consumers were more likely to have a lower BMI.

Although there is a paucity of research to date on the consumption levels of LCS in pregnant women, a study including pregnant women in the USA indicated that approximately a quarter of women consumed LCS during pregnancy, with this being the case across the trimesters [2]. To our knowledge, there have been no studies in Europe to assess LCS intake in pregnant women, thus our results are important to contribute to this gap. Intakes have been reported in adult populations in Australia [25], the USA [6] and the UK [26] with 18%, 30% and 32% of adults being classified as consumers, respectively. These figures are similar to our findings for a pregnant cohort in Ireland. An understanding of LCS consumption is necessary to subsequently determine how these intakes are associated with offspring health and may be important for fetal programming. LCS have been found to be associated with changes in maternal gut microbiome [27], which is important for determining infant microbiome [12, 28].

In the Western diet, the main sources of LCS have been suggested to be beverages and table-top sweeteners [15]. The most commonly consumed LCS-containing food group amongst the ROLO cohort of pregnant women was diet cola, with this being the case for each trimester. There were also relatively high numbers of consumers of low-calorie fruit drinks and low-calorie yogurts in this cohort. There was little or no consumption of the other food groups. This is similar to research in the general population which shows carbonated soft drinks and yogurts to be the LCS-containing products which were consumed in the largest quantities [25, 29]. Intakes of LCS in an Irish adult population have been investigated in the National Adults Nutrition Survey using data collected in 2011. This indicated that sauces were the most commonly consumed LCS-containing food, with energy reduced and no added sugar carbonated flavoured drinks and dairy products also frequently consumed in the general Irish adult population [30]. Our data were also collected between 2007 and 2011 indicating that consumption is consistent in pregnant and non-pregnant populations in Ireland. Similarly, more than a quarter of pregnant women in the Canadian Healthy Infant Longitudinal Development (CHILD) Study were consumers of artificially sweetened beverages [13]. In the ROLO cohort, approximately 20% of the total cohort included in the current analysis were consumers of diet cola, which is slightly lower than in Canada.

There were significantly more consumers of LCS in the intervention arm of the study than in the control group in trimester 2, which coincided with the commencement of the intervention. Participants in the intervention group received dietary advice for a low-GI diet as part of the study, therefore it is likely that they have increased consumption of LCS-containing products due to a low-GI diet discouraging consumption of sugar-sweetened beverages and sugary foods as they are classified as having a high GI. There were no significant differences in dietary intake of any of the food groups between the intervention and control group in any of the trimesters, except for higher intake of low-calorie yogurts in the intervention group in trimester 1. This trimester was before the intervention study began, and so it suggests those in the intervention group were consumers of low-calorie yogurts before they received advice on following a low-GI diet. It is important to note that the dietary data included in the current study were collected approximately 10 years ago, between 2007 and 2011. Given that a sugar-sweetened drinks tax was introduced in Ireland in 2018 [31], it is possible that the consumption of LCS has increased in the Irish population in recent years.

The current study has many strengths. First, the large sample size of the ROLO study and detailed dietary data collected during each trimester of pregnancy is a particular strength, allowing for LCS consumption across the trimesters to be determined. This also allowed us to determine if a woman who was initially a consumer remained a consumer for the duration of pregnancy. Previous research published to date is largely cross-sectional, and thus does not indicate whether individual consumption habits change over time. Also, the use of a food diary is thought to be representative of daily intake, providing more detail of the amounts and types of foods actually eaten than other methods of dietary data collection such as food frequency questionnaires [32]. This is the first study to investigate LCS consumption in pregnant women in Ireland, and to the best of our knowledge this has not been reported previously for other European populations. The current analysis also determined which foods and beverages contain LCS, whereas some previous studies have included beverages only. These results also allow for an insight into how a low-GI dietary intervention can influence LCS consumption. Given that we have shown that consumers were more likely to be in the intervention group, it is suggestive that the change in sugars to sweeteners as a result of a low-GI diet is evident here and thus a low-GI diet has an impact on consumption levels of LCS. There are some limitations to the study. The dietary data analysis software does not give intakes of individual sweeteners; therefore, we have determined which foods may contain LCS and subsequently created appropriate food groups for the current analysis. Dietary analysis software which allows data on consumption of individual LCS would be an advantage. Also, low-calorie yogurts were identified as an LCS-containing food group, however, it is possible that not all low-calorie yogurts contain LCS as some may have low fat but in fact have additional sugars added. The consumption of table-top sweeteners was not recorded in this study which is a further limitation.

To conclude, approximately a third of pregnant women were found to be consumers of LCS. A low-GI dietary intervention impacted consumption of LCS during pregnancy with consumption of LCS-containing foods more common in those who were following a low-GI diet. Future work could focus on estimating exposure to LCS using maximum permitted level data for each of the sweeteners included which would provide an estimate of actual consumption and exposure to each sweetener. The influence of prenatal exposure to LCS on pregnancy and child outcomes at various ages warrants further investigation.

## Supplementary information

Supplementary data
