# Supplementary material for: The consumption of low-calorie sweetener containing foods during pregnancy: results from the ROLO study
Source: Eur J Clin Nutr. 2021 May 26;76(2):227–34. doi: 10.1038/s41430-021-00935-0 (PMC8821016; doi:10.1038/s41430-021-00935-0)
Supplement: Supplementary file 1 — Supplementary data [file 41430_2021_935_MOESM1_ESM.docx]

**Supplementary table 1:** Percentage of pregnant women who were consumers in trimester 1, and percentage who remained consumers or changed their consumption

|  | **Intervention** | | **Control** | |
| --- | --- | --- | --- | --- |
|  | **n** | **%** | **n** | **%** |
| **Total participants** | 258 |  | 313 |  |
| **Consumers trimester 1^a^** | 74 | 28.7 | 86 | 27.5 |
| **Remained consumers^b^** |  |  |  |  |
| **Trimester 2** | 53 | 71.6 | 50 | 58.1 |
| **Trimester 3** | 40 | 54.1 | 36 | 41.9 |
| **No longer consumers^c^** | |  |  |  |
| **Trimester 2** | 21 | 28.4 | 36 | 41.9 |
| **Trimester 3** | 13 | 17.6 | 14 | 16.3 |
| **Became consumers^d^** |  |  |  |  |
| **Trimester 2** | 49 | 19.0 | 27 | 8.6 |
| **Trimester 3** | 17 | 6.6 | 34 | 10.9 |
| **Never consumers^e^** | 118 | 45.7 | 166 | 53.0 |
| *^a^as % of total participants  ^b^remained consumers from trimester 1, percentage as % of consumer in trimester 1  ^c^no longer consumers from trimester 1, percentage as % of consumers in trimester 1 ^d^became consumers who were not consumers in trimester 1, as % of total participants  ^e^were never consumers in any trimester, as % of total participants* | | | | |
